# Rapid Progression to Complete Paraplegia After Electroacupuncture in a Patient With Spinal Dural Arteriovenous Fistula: A Case Report

**DOI:** 10.3389/fsurg.2021.645884

**Published:** 2021-08-27

**Authors:** Ki-Hoon Park, Chang-Hoon Jeon, Nam-Su Chung, Han-Dong Lee

**Affiliations:** Department of Orthopaedic Surgery, Ajou University School of Medicine, Suwon, South Korea

**Keywords:** spinal dural arteriovenous fistula, paraplegia, neurologic deficits, electroacupuncture, spinal vascular malformation

## Abstract

Spinal dural arteriovenous fistula (SDAVF) usually has an insidious clinical course, but 5–15% of the cases have acute exacerbations. In some cases, there is an abrupt progression to paraplegia following an epidural injection or anesthesia. Electroacupuncture is a form of acupuncture that applies a small electrical current to needles inserted at specific points in the body. It is widely used for its analgesic effect on back pain. In this study, we report a rare case of SDAVF in which the symptoms of a patient worsened rapidly to complete paraplegia within a few hours after applying electroacupuncture to his back. A 49-year-old man had rapid progression to complete paraplegia within a few hours of electroacupuncture on his back. MRI showed SDAVF and worsening of cord signal change. An emergency operation was performed to ligate the SDAVF. The patient was able to walk 1 month post-operatively. Most of the neurological deficits had disappeared by 1 year post-operatively, with normalization of MRI. Our case emphasizes that SDAVF patients should be careful when exposed to any circumstances that might affect the circulation around the dural arteriovenous fistula, such as electroacupuncture. Patients should also be warned in advance about the possibility of rapid exacerbation of neurological symptoms. Regardless of the severity of the neurological symptoms, immediate treatment is essential for recovery and a better outcome.

## Background

Spinal dural arteriovenous fistula (SDAVF) is a vascular malformation of the spinal cord and dura mater consisting of a shunt between the dural branch of the radicular artery and the vein on the dura mater (1). It is rare but accounts for 80% of all spinal arteriovenous malformations (1). Usually, it is diagnosed after the initiation of neurologic symptoms, but the functional outcome after treatment is generally favorable (2). The average time from symptom onset to diagnosis is about 23 months, due to the slow progression of the disease (3). However, there have been some cases reporting abrupt progression even to paraplegia in SDAVF patients, following epidural injection or anesthesia (4–6).

Electroacupuncture is a form of acupuncture based on the concepts of traditional Chinese medicine and is currently widely used for its analgesic effect on chronic pain disorders, such as back pain. It works by applying a small electrical charge to the needles inserted into specific points along the body bringing about transcutaneous electrical nerve stimulation (7). In this study, we introduce a rare case of surgically treated SDAVF in which the symptoms of a patient rapidly aggravated within a few hours after applying electroacupuncture on his back.

## Case Presentation

**Figure 1:**
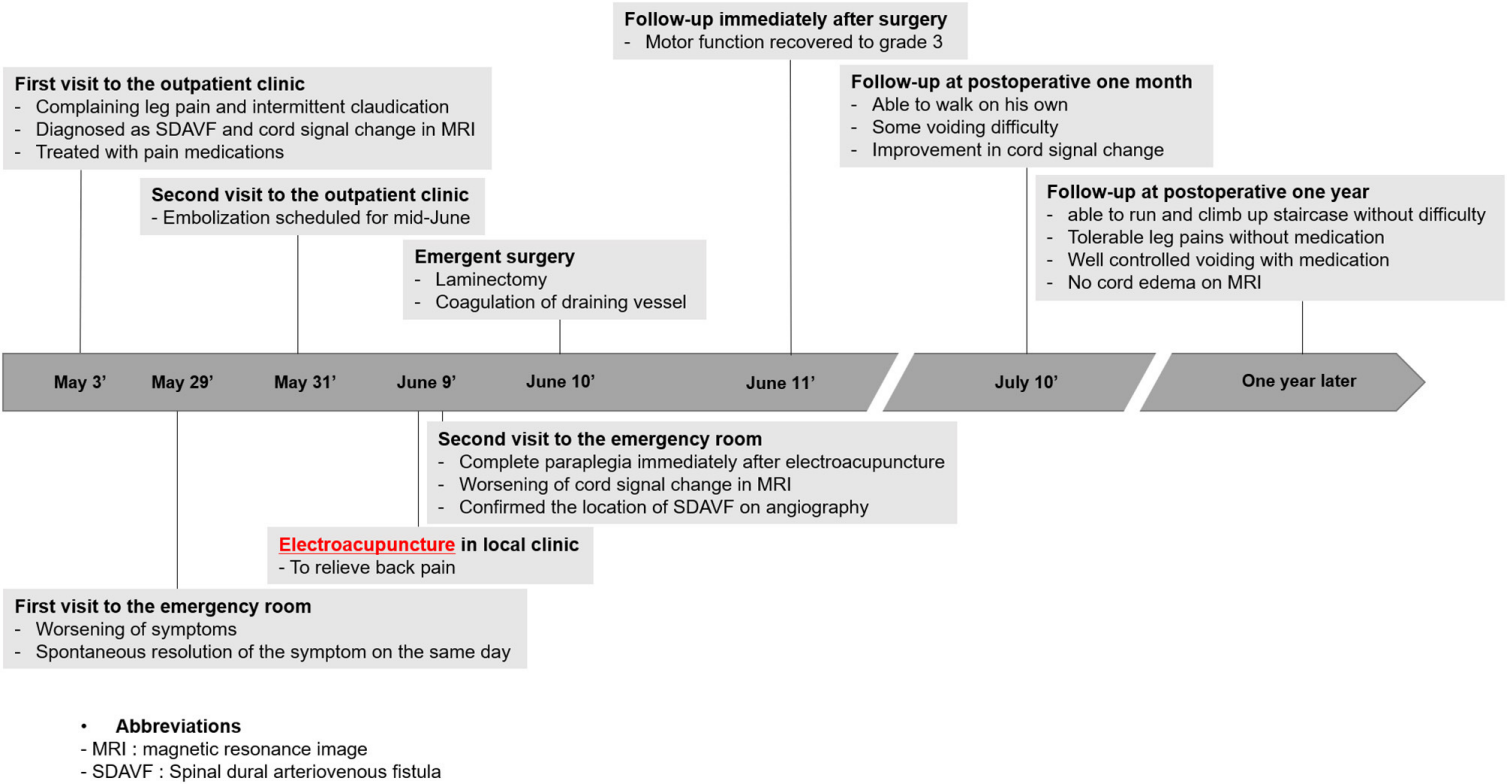
Timeline of the current case.

**Figure 2:**
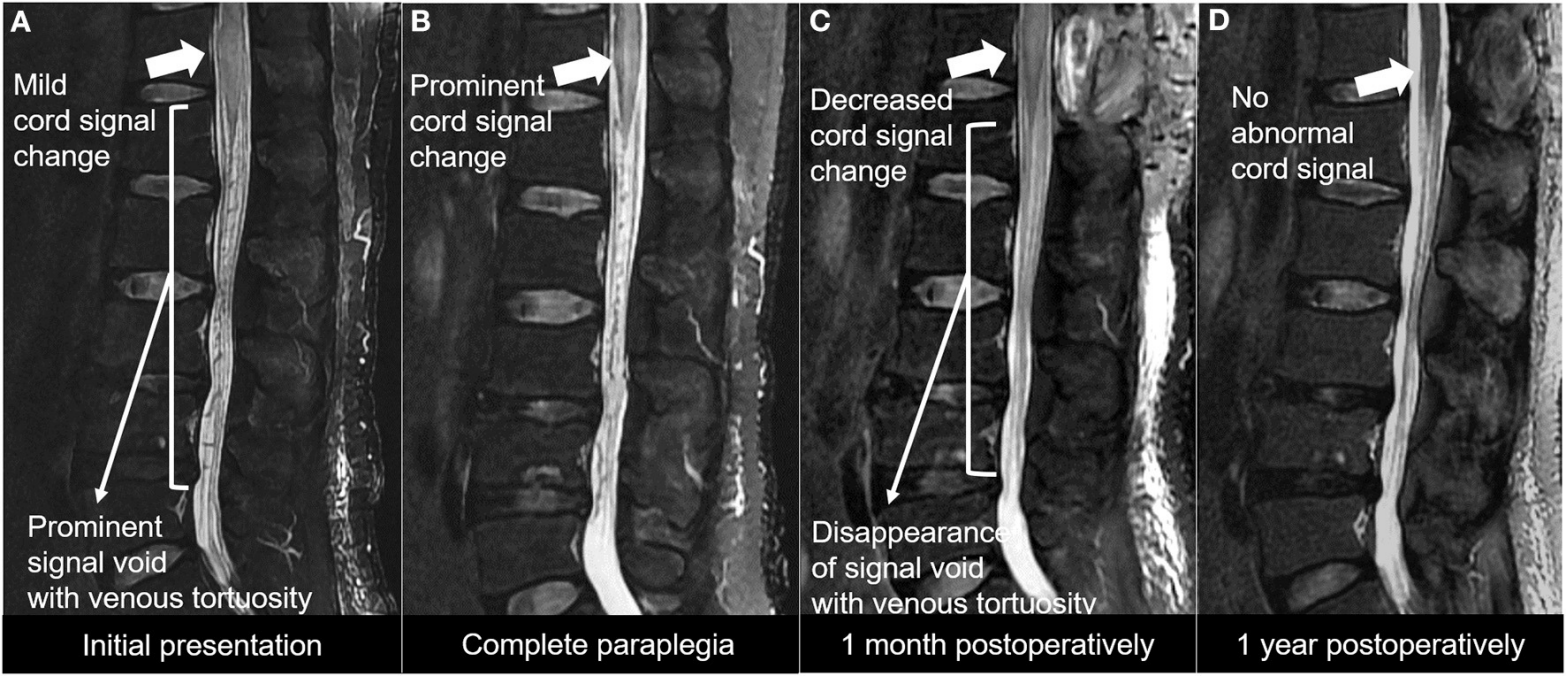
**(A)** T2-weighted MRI study showing prominent signal void with redundant venous tortuosity, which represents spinal arteriovenous fistula. **(B)** T2-weighted MRI study showing worsening of spinal cord signal change. **(C)** T2-weighted MRI study at post-operative 1 month showing decreased cord signal change and disappearance of the signal void with redundant venous tortuosity. **(D)** T2-weighted MRI study at post-operative 1 year no abnormal cord signal change.

The whole timeline of the current case is depicted in Figure 1. A 49-year-old male visited our outpatient clinic complaining of both lower extremities radiating pain persisting over 2 months. He had no past medical history. He was 185 cm tall, weighed 93 kg, and was slightly overweight with a BMI of 27.2. The patient reported subjective leg weakness, especially while walking. Physical examination showed intact motor and sensory, non-specific femoral nerve stretching test and straight leg raise test, but Babinski sign was positive. Initial modified Aminoff and Logue's scale (ALS) (8) was 2 (restricted exercise tolerance), 0 (normal), and 0 (normal) for gait, urine, and defecation, respectively. Initial MRI study from another hospital showed cord signal change and redundant intradural venous tortuosity, which were typical findings of SDAVF (Figure 2A). Drugs were prescribed to alleviate symptoms, and the course and the likelihood of exacerbation of SDAVF were explained.

After 2 weeks from the initial outpatient clinic visit, the patient came to our emergency department with a back sprain followed by a worsening of radiating pain and weakness. He was discharged without further evaluation as his symptoms were relieved spontaneously and there was no residual neurological abnormality.

**Figure 3:**
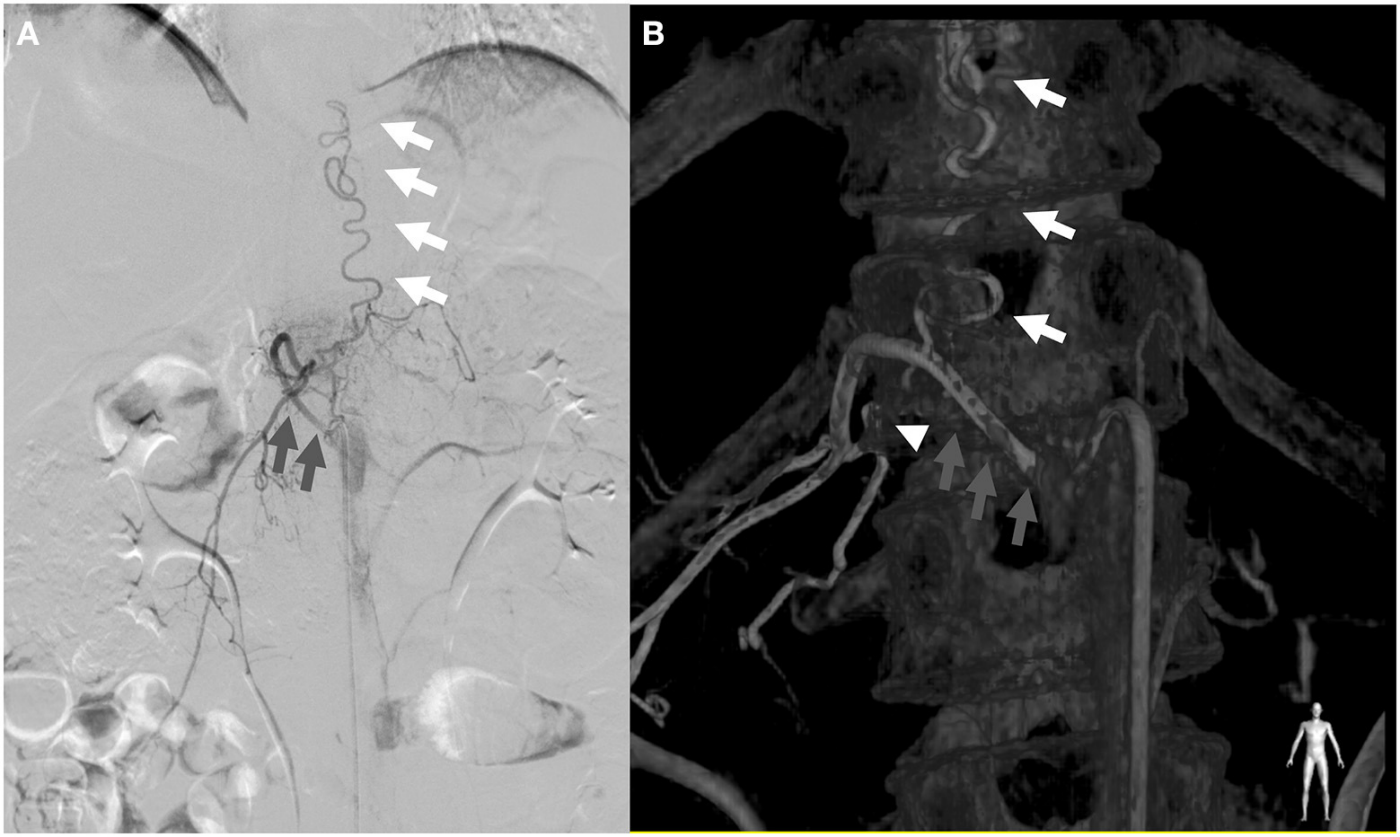
Angiogram **(A)** of the right T12 segmental artery and three-dimensional (3D) reconstructed image **(B)** demonstrating the main arterial feeder of the radiculomengingeal artery (white arrowhead) arising from the right T12 segmental artery (gray arrow) connected to the engorged perimedullary vein (white arrow).

Two days later, he visited the outpatient clinic again and we decided to perform the endovascular treatment. However, 5 days after the second outpatient clinic visit, while waiting for the endovascular intervention schedule, the patient visited our emergency room again with both lower extremity motor weakness of grade 0 from both hip and toe and a sensory deficit of 90% in both lower extremities. He reported that his symptoms started after applying electroacupuncture on his back. It was recorded in the medical record that no other drugs were injected during the electroacupuncture procedure. The anal tone was decreased, and the perianal sensory deficit was also seen. ALS deteriorated to 5 (requires a wheelchair), 3 (persistent incontinence or retention), and 3 (persistent incontinence) for gait, urine, and defecation, respectively. MRI study showed worsening of cord signal change compared with the initial exam (Figure 2B). Angiography revealed SDAVF with a feeding artery from the right segmental artery of T12-L1 forming fistula near neural foramen and engorgement of perimedullary vein running vertically (Figure 3). The intervention team was unable to schedule an emergency procedure, so we chose surgical treatment.

**Figure 4:**
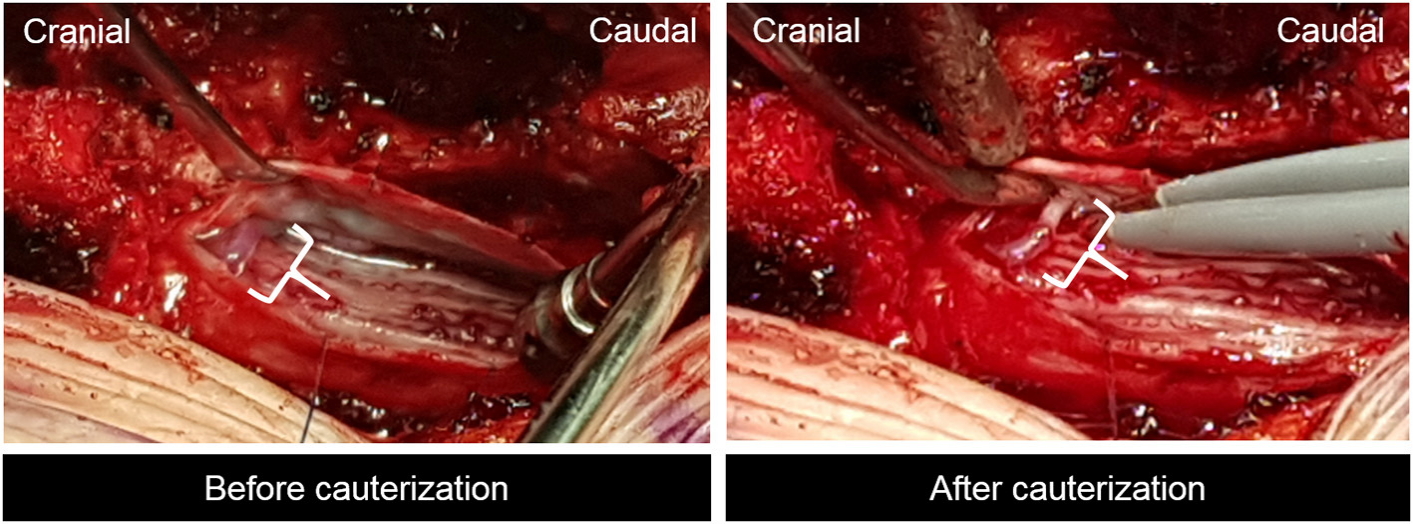
Intraoperative finding revealing intradural arteriovenous fistula.

We performed laminectomy of the T12 vertebra, and an incision was made on the dural sac. SDAVF was found and obliterated with a bipolar coagulation unit (Figure 4). After surgery, motor function recovered to grade 3 at the immediate post-operative period and continued to improve over time. At post-operative 1 month, the patient was able to walk on his own. ALS improved to 2 (restricted exercise tolerance), 2 (occasional incontinence or retention), and 2 (occasional incontinence or persistent constipation) for gait, urine, and defecation, respectively. Follow-up MRI showed improvement in cord signal change (Figure 2C). At post-operative 1 year, the patient was able to run and climb up the staircase without difficulty. Leg pains were well-controlled without medications. He had some voiding difficulty before, but it was well-controlled with medication. ALS score improved to 0 (normal), 1 (urgency, frequency, and/or hesitancy), and 1 (mild constipation, responding well to aperients) for gait, urine, and defecation, respectively. There was no recurrence of neurologic deficits. MRI performed at 1-year follow-up showed no abnormal cord edema or abnormality (Figure 2D). The patient was satisfied with the outcome of the surgery.

The patient provided written informed consent to publish the information including MRI images.

## Discussion

The most common early symptoms of SDAVF include gait disturbance, paresthesia, and sensory deficit (9, 10). In most cases, neurological symptoms progressed slowly, leading to complete paraplegia, and intermittent remissions are also reported (11). Due to its obscure clinical manifestations, a significant delay is often present before the diagnosis of SDVAF is made (9). Once SDAVF is confirmed, treatment is essential because if untreated, the lesion can cause significant neurological disability, and spontaneous closure is extremely rare (12). Treatment options include surgical ligation and endovascular embolization. Endovascular treatment is less invasive, but in cases when the malformation vasculature is in proximity to the anterior or posterior spinal artery or artery of Adamkiewicz, embolization may cause spinal ischemia and at that point surgical treatment is needed (13). Surgical treatment is to ligate the draining vein, and this is proven to be as effective as totally removing the draining vein (14).

Spinal dural arteriovenous fistula usually shows insidious and progressive clinical course, but 5–15% of the cases show acute myelopathic exacerbation (15). There are not many reports of such acute exacerbation in SDAVF patients. Our case showed an uncommon rapid deterioration to complete paraplegia with sphincter dysfunction just in several hours. It was a unique case in which the symptoms deteriorated rapidly after applying electroacupuncture on the back of the patient. To the extent of our knowledge, there were no such cases reporting the effect of electroacupuncture in SDAVF patients and no such cases showing complete paraplegia after applying electroacupuncture.

In general, the spinal arteries have a higher blood pressure than the spinal veins (normal pressure gradient), and oxygen and nutrients are supplied to the spinal cord through capillaries. However, if an acute exacerbation occurs in SDAVF, venous pressure increases (pressure gradient change), resulting in decreased blood supply from the spinal arteries through the capillaries to the veins, thereby causing vascular congestion with intramedullary edema and ischemia (11). The most common cause of pressure gradient change is an increase in intrathecal pressure. Therefore, it has been reported that the symptoms worsened in patients with SDAVF in the prone position for a long time during spinal surgery (16, 17), or after singing or standing for a long time (18, 19). Electroacupuncture is a method of applying electrical stimulation with about 20 needles inserted in the prone position for about 20–30 min (20), and it is highly likely that the increase in intrathecal pressure caused by the prone position for a long time made the condition worse. In particular, since the patient was slightly overweight, this may have contributed to the worsening of his condition.

The second hypothesis for the deterioration of the condition of the patient is the pressure gradient change due to direct damage by acupuncture needles or electric stimulation. In the previous studies, there have been reports of CSF leaks or direct damage to the spinal cord caused by acupuncture needles (21, 22). But in the current case, no CSF leak or direct damage to the spinal cord was observed in the surgical field. However, one of the positions of acupuncture needles (GV-3) for backpain treatment in electroacupuncture is between the L3 and L4 spinous processes (20). As in the previous cases, the deeper puncture may have affected the thecal sac and the spinal cord. There have been reports of peripheral nerve damage or dysfunction of implantable cardioverter–defibrillators due to electrical stimulation of electroacupuncture (23). The deeper position of the electroacupuncture needle may have inflicted direct damage to the thecal sac or nerve. Also, it has been reported that electrical stimulation using needles can change the microcirculation of surrounding tissues (24). In the course of treating the back pain of this patient using electroacupuncture, the possibility that the electrical stimulation needle located around the spinal canal changed microcirculation and caused the pressure gradient change cannot be excluded.

Our case presented with a severe neurological deficit of complete paraplegia with sphincter dysfunction. Studies show that long duration of symptoms before treatment and severe pre-operative neurological deficit adversely affect the long-term outcome (25–27). Only about one-third of the patients who were wheelchair-bound by the time of diagnosis of SDAVF were able to ambulate with a walker 90 days after treatment (28). The previous studies have reported that ALS for gait, urination, and defecation recovered approximately 1 point each at post-operative 1 year (8, 29). In the case of the most severe disability in each item, it was very rare that the patient recovered close to normal (30). However, just as in our case, reversal of myelopathy is possible with prompt treatment before irreversible neurological damage is done (31). The patient from our case showed rapid recovery, being able to ambulate without assistance at post-operative 1 month. As the second hypothesis, we speculated that electrical stimulation-induced neurological deterioration may be related to the reversal of paraplegia.

## Conclusions

Our case was unique for its unusual development after applying electroacupuncture, not only to mention the rapid progression of myelopathic symptoms in just a few hours but also to the severity to the extent of complete paraplegia, all of which resolved quickly over time after prompt surgical treatment. Therefore, our case presents that SDAVF patients should take caution while being exposed to any circumstances, such as electroacupuncture, that might affect the circulation around the SDAVF. Also, patients should be warned in advance about the possibility of rapid exacerbation of neurologic symptoms, and regardless of the severity of neurological symptoms, immediate treatment is essential for recovery and better outcome. Although the recovery process in this case was good, since it was a single case, the results may not necessarily be good. Further research studies are needed in the future.

## Data Availability Statement

The original contributions presented in the study are included in the article/supplementary material, further inquiries can be directed to the corresponding author.

## Ethics Statement

Written informed consent was obtained from the patient for the publication of this case report.

## Author Contributions

C-HJ and N-SC conceived the idea and conceptualized the study. K-HP, C-HJ, and H-DL collected and analyzed the data. K-HP and H-DL drafted the manuscript. C-HJ and N-SC reviewed the manuscript. All authors read and approved the final draft.

## Conflict of Interest

The authors declare that the research was conducted in the absence of any commercial or financial relationships that could be construed as a potential conflict of interest.

## Publisher's Note

All claims expressed in this article are solely those of the authors and do not necessarily represent those of their affiliated organizations, or those of the publisher, the editors and the reviewers. Any product that may be evaluated in this article, or claim that may be made by its manufacturer, is not guaranteed or endorsed by the publisher.
